# New insights into cheddar cheese microbiota-metabolome relationships revealed by integrative analysis of multi-omics data

**DOI:** 10.1038/s41598-020-59617-9

**Published:** 2020-02-21

**Authors:** Roya Afshari, Christopher J. Pillidge, Elizabeth Read, Simone Rochfort, Daniel A. Dias, A. Mark Osborn, Harsharn Gill

**Affiliations:** 1 School of Science, RMIT University, Bundoora, PO Box 71, Bundoora, VIC 3083 Australia; 2 School of Health and Biomedical Sciences, RMIT University, Bundoora, PO Box 71, Bundoora, VIC 3083 Australia; 3 Biosciences Research Division, Department of Environment and Primary Industries, AgriBiosciences, 5 Ring Road, Bundoora, Victoria VIC 3083 Australia

**Keywords:** Metabolomics, Metagenomics

## Abstract

Cheese microbiota and metabolites and their inter-relationships that underpin specific cheese quality attributes remain poorly understood. Here we report that multi-omics and integrative data analysis (multiple co-inertia analysis, MCIA) can be used to gain deeper insights into these relationships and identify microbiota and metabolite fingerprints that could be used to monitor product quality and authenticity. Our study into different brands of artisanal and industrial cheddar cheeses showed that *Streptococcus*, *Lactococcus* and *Lactobacillus* were the dominant taxa with overall microbial community structures differing not only between industrial and artisanal cheeses but also among different cheese brands. Metabolome analysis also revealed qualitative and semi-quantitative differences in metabolites between different cheeses. This also included the presence of two compounds (3-hydroxy propanoic acid and O-methoxycatechol-O-sulphate) in artisanal cheese that have not been previously reported in any type of cheese. Integrative analysis of multi-omics datasets revealed that highly similar cheeses, identical in age and appearance, could be distinctively clustered according to cheese type and brand. Furthermore, the analysis detected strong relationships, some previously unknown, which existed between the cheese microbiota and metabolome, and uncovered specific taxa and metabolites that contributed to these relationships. These results highlight the potential of this approach for identifying product specific microbe/metabolite signatures that could be used to monitor and control cheese quality and product authenticity.

## Introduction

Cheese microbiota play a pivotal role in the development of cheese flavor and the product quality and safety of cheese. These attributes can be achieved by tightly controlled manufacturing conditions, excellent hygiene and use of commercial starter cultures (usually strains of *Lactococcus lactis*). Following manufacture, when starter bacteria cease to dominate, cheese must be ripened for full flavour development. This is a highly complex process, with successions within microbial communities, and their associated enzymes and biochemical reactions occurring over time to release numerous flavoursome organic compounds^1^. Ripening can be controlled to some extent by the addition of known adjunct cultures, usually strains of lactobacilli, but adventitious bacteria from the factory environment also contribute to the ripening process and to the composition of the cheese microbiota^2^.

Advances in DNA sequencing using DNA extracted directly from samples have enabled improved understanding of the microbial communities found in cheese, and especially of less-abundant microorganisms which can nevertheless have significant impacts on cheese flavour and safety^3,4^. Although microbial communities vary depending on the cheese type, in hard cheeses such as cheddar that undergo ripening, after the starter bacteria die off lactobacilli are dominant, and may be found in combination with a number of other genera not belonging to the lactic acid bacteria group – for example coagulase-negative staphylococci and Actinobacteria^5–7^. Little is known about the associations between important flavour compounds in these cheese types and the many different bacterial and/or fungal species that arise over time during cheese ripening.

Analysis of metabolites in ripened cheese can be used to identify important flavour compounds (e.g. volatile compounds) and indirectly show which types of bacteria are likely to be present. This includes both untargeted and targeted chemical analyses to detect specific metabolites or flavour precursor compounds such as amino acids and organic acids^8–10^. A more complete understanding of cheese ripening can now be gained through improvements in analytical techniques such as mass spectrometry combined with untargeted chemical analysis and systems to handle these very large datasets, with some studies indicating the presence of hundreds or thousands of compounds including many not previously reported in cheese^11^.

Despite these advances, the interrelationships between cheese microbiota and their metabolites remain largely unstudied. This type of information could be useful if translated into cheesemaking practice; for example, if ripening conditions or application of adjuncts were adjusted to favour the growth of specific microorganisms associated with production of desirable flavour compounds. To better understand these interactions, integrative analysis of large multiple-omics datasets can now be applied, facilitated by the application of new computational algorithms and high-end computing systems, as we have previously advocated^12^. Tools that facilitate the integration of multiple omics data sets are now becoming increasingly available^13^.

In this study we have carried out an integrative analysis to investigate interrelationships between cheese microbiota and cheese metabolomes in artisanal and industrial cheddar cheeses, using a combination of 16S rRNA-based microbiota analysis and untargeted metabolomics analyses using LC-MS/MS and GC-MS with data integration via multiple co-inertia analysis (MCIA). MCIA is a multivariate analysis technique that allows systematic integration of more than two omics datasets; it identifies co-relationships between multiple high-dimensional datasets and explores the variance from within each dataset to facilitate biological interpretation and pathway analysis^14^. We chose two industrial and two artisanal cheddar cheeses and additionally compared microbiota and metabolomes at the cheese surface, which is relatively aerobic, and within the cheese (core samples) which are highly anaerobic. This is the first time to our knowledge that such integrative analysis has been applied to a complex ecosystem in a fermented food.

## Results

### Variation and composition of the cheese microbiota

**Figure 1:**
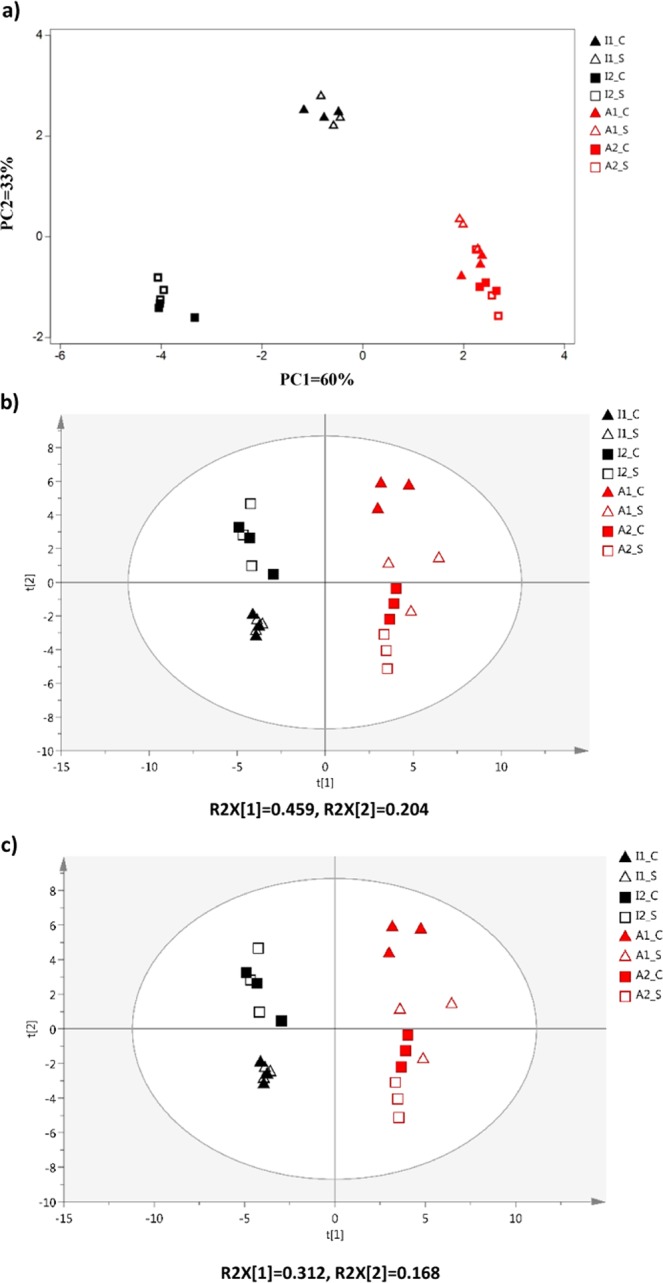
Principal component analysis (PCA) of industrial and artisanal cheeses for (**a**) microbiota, (**b**) GC-MS metabolites and (**c**) LC-MS metabolites. I: industrial, A: artisanal; C: core, S: Surface; number indicates different brand for each artisanal and industrial cheese.

Sequencing of PCR-amplified 16S rRNA gene amplicons targeting the V4-region from total DNA revealed 159 operational taxonomic units (OTUs) binned at 97% identity and a threshold of detection of an OTU being present in more than one sample (overall). Principal component analysis (PCA) and analysis of similarities (ANOSIM) based on a Bray Curtis dissimilarity matrix, showed significant differences between the structure and composition of the bacterial communities between the industrial and artisanal cheeses (R = 0.94, significance level = 0.1%) and also between communities from the two industrial brands (R = 0.94, significance level = 0.1%) (Fig. 1a). Conversely, microbiota composition did not differ between the artisanal brands (R = 0.62, significance level = 0.6%) or between core and surface samples (R = 0.64, significance level = 0.5%).

**Figure 2:**
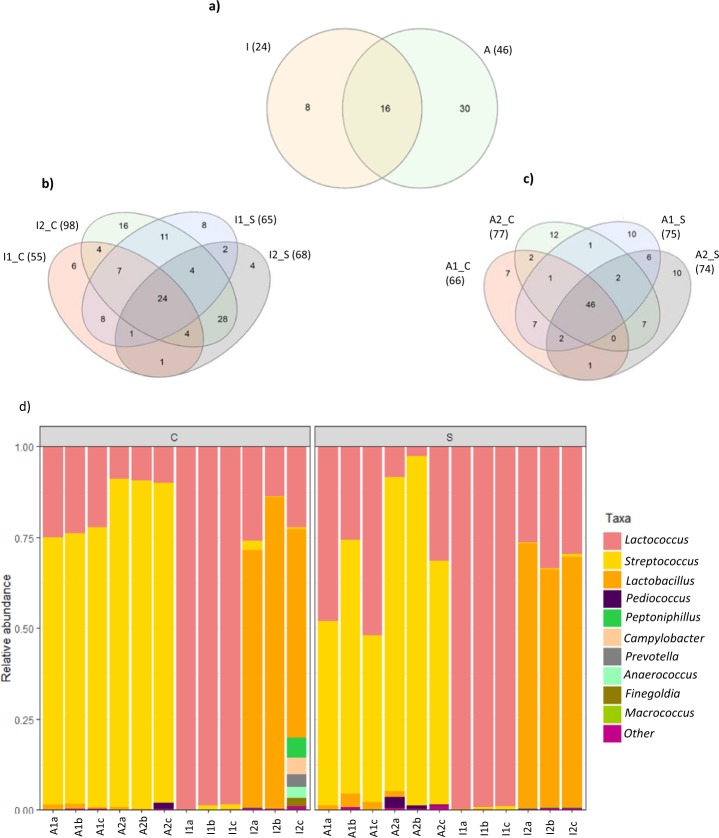
Venn diagram representing the unique and shared OTUs between all cheeses (**a**), between industrial cheeses (**b**) and between artisanal cheeses (**c**). Microbiota composition of the cheddar cheeses at genus level (**d**). The ten most abundant genera are shown. I: industrial, A: artisanal; C: core, S: Surface; a-c: sample replicates, 1–2: different brands within each artisanal and industrial cheese.

Of the 159 OTUs, sixteen OTUs were present in all industrial and artisanal cheeses with these OTUs representing more than 70% of the relative abundance of bacterial taxa found in the samples (Fig. 2a). These sixteen OTUs were assigned to the genera *Streptococcus*, *Lactococcus* and *Lactobacillus*. Overall, twenty-four OTUs were present in all industrial cheeses (Fig. 2b) and forty-six OTUs were common to all artisanal samples (Fig. 2c). Shared OTUs in industrial cheeses were assigned to *Streptococcus*, *Lactococcus*, *Lactobacillus* and in artisanal cheeses OTUs were assigned to *Streptococcus*, *Lactococcus*, *Lactobacillus*, *Staphylococcus* and non-classified taxa.

The prevalence of the ten most abundant genera present within cheese samples is depicted in Fig. 2d. The most abundant genera in industrial cheeses for both core and surface samples were either *Lactococcus* or *Lactobacillus* depending on the origin of manufacture. However, *Streptococcus* (presumably mostly *S. salivarius* subsp*. thermophilus*) followed by *Lactococcus* and *Lactobacillus* were the most abundant genera in artisanal cheeses for both core and surface samples. Other genera were present at much lower relative abundances (the relative abundance <1%) in both core and the surface samples of industrial cheeses namely, *Streptococcus* and *Macrococcus*. However, in one of the artisanal cheese brands *Pediococcus* was present at low abundance in both core and surface samples.

### Cheese GC/MS metabolome profiling

GC-MS untargeted metabolomics identified a total of 46 metabolites in which the chemical identity could be confirmed by comparison of their retention times and mass spectra data to either *in-house* libraries and/or authentic standards. These included 22 amino acids or amines (AA); 18 organic acids (OA) and fatty acids (FA) or sterols; and 6 sugars or sugar phosphates (Supplementary Table S1). PCA of GC-MS metabolome data revealed that industrial and artisanal cheeses were separated along PC1 (Fig. 1b). Similarly, each brand of artisanal or industrial cheese was separated along PC2 and for one of the artisanal brands, core and surface metabolomes were also separated.

Differences between metabolite response ratios between artisanal and industrial cheeses was determined by pairwise *t*-test with fold changes (in the abundance of metabolites) presented as being relative to abundance in industrial cheeses (Supplementary Table S1). Six compounds (GABA, 5-amino-valeric acid, tyramine, 3-hydroxypropanoic acid, galactonic acid and glutaric acid) were found to be present only in the artisanal cheeses. Succinic acid was found to be present at significantly higher abundance in artisanal cheeses in both core and surface samples. However, free fatty acids including heptadecanoic acid, pentadecanoic acid, octadecanoic and decanoic were significantly higher in their relative abundance only in the surface of artisanal cheeses when compared to industrial cheeses. Conversely, urea and lysine were both present at a significantly higher relative abundance in industrial cheeses relative to the artisanal cheeses.

### Cheese LC/MS metabolome profiling

LC-MS untargeted metabolomics analysis revealed over 8000 features in the cheddar cheeses (4010 in positive ESI mode and 4100 in negative ESI mode). The 1000 most dominant peaks were chosen for further analysis. Putative IDs of features were determined using the Human Metabolome Database and METLIN. Given the large number of metabolites present, we did not attempt to determine IDs of all the peaks but only those that were significantly different between cheeses or that were found to have significant associations with the microbiota data. When LC-MS metabolites were subjected to PCA analysis, consistent with GC-MS metabolome profile, industrial and artisanal cheeses were again separated along PC1 and each artisanal or industrial brand was separated along PC2 (Fig. 2c). In contrast to the GC-MS metabolome profiles, core and surface of artisanal cheeses were separated along PC1; however, separation was not observed between the core and the surface metabolomes of industrial cheeses.

Table 1 shows the statistical significance of differential LC-MS-metabolites levels generated using a volcano plot along with corresponding effect size and their tentative metabolite identity for core and surface samples. Among 1000 features, the abundance of six and nine features differed significantly between industrial and artisanal cheese for core and surface samples, respectively (Table 1). Four compounds in the core of industrial cheeses including one amino acid (arginine) and one dipeptide and five compounds in the surface of industrial cheeses including dipeptides had significantly higher abundances when compared to artisanal cheeses (*P value* < 5*10^−5^). However, two and four compounds were present in significantly higher abundances in the core and surface samples of artisanal cheeses when compared to industrial cheeses. The most pronounced differences between industrial and artisanal cheeses was the higher proportion of the nitrogenous compound (putatively identified as butyrobetanine) in artisanal cheese compared to industrial cheeses. A putatively identified phenolic compound (*O*-methoxycatechol-*O*-sulphate) was more abundant in the surface of artisanal cheeses compared to industrial cheeses. However, the identities of five features which differed significantly between cheeses could not be determined due to the low abundance of associated peaks and inability to capture adequate MS-MS data.

**Table 1:** LC/MS features that differed significantly between industrial and artisanal cheeses and their associated taxon (q < 0.1).

	M/Z	RT	putative identity	Directed Effect Size [ART vs. IND]	OTU	Taxon	Association
Core	146.1177	1.27	gamma-Butyrobetaine	1.06E + 08	260	*Streptococcus_salivarius*	Positive
	279.16	10.92	ND	277931.66	260	*Streptococcus_salivarius*	Positive
	361.20	11.8	ND	−323616.7	167	*Streptococcus_salivarius*	Positive
	214.05	1.18	ND	−5361950	98	*Streptococcus_salivarius*	Negative
	173.1	1.15	*Arginine	−3.46E + 07	41	*Streptococcus_porcinus*	Negative
	232.1293	1.28	ASN-VAl	−1.72E + 08	2	*Streptococcus_salivarius*	Negative
Surface	146.117	1.27	gamma-Butyrobetaine	4.58E + 07	72	*Streptococcus_salivarius*	Positive
	103.038	2.65	Unknown	2.62E + 07	174	*Streptococcus_porcinus*	Positive
	242.012	3.51	Unknown	2.57E + 07	151	*Streptococcus_salivarius*	Positive
	203.001	5.28	O-methoxycatechol-O-sulphate	1.37E + 07	174	*Streptococcus_porcinus*	Positive
	119.025	15.53	ND	−8220653.5	72	*Streptococcus_salivarius*	Negative
	247.093	1.27	Threoninyl-Glutamate	−2.91E + 07	72	*Streptococcus_salivarius*	Negative
	258.145	1.97	Di peptide containing glutamate	−4.12E + 07	167	*Streptococcus_salivarius*	Negative
	187.108	1.99	ND	−4.32E + 07	167	*Streptococcus_salivarius*	Negative
	231.134	2.014	Unknown	−1.33E + 08	—	—	—

Data represents directed effected size (artisanal cheese vs industrial cheeses) for each cheese obtained from volcano plot and significant at *P-value* ≤ 0.0001 (*Bonferroni corrected P value*). *Metabolite which their putative IDs is validated by comparison of their RT and *m/z* to an external standard. ND: not detected due to the low abundance of associated peaks and inability to capture adequate MS2 data. See Supplementary Table S3 for MS2 data associated with each peak. Those correlation between each metabolite and taxon which were significant at *q value* < 0.1 were shown.

### Integrative analysis of cheese microbiome and metabolome datasets

**Figure 3:**
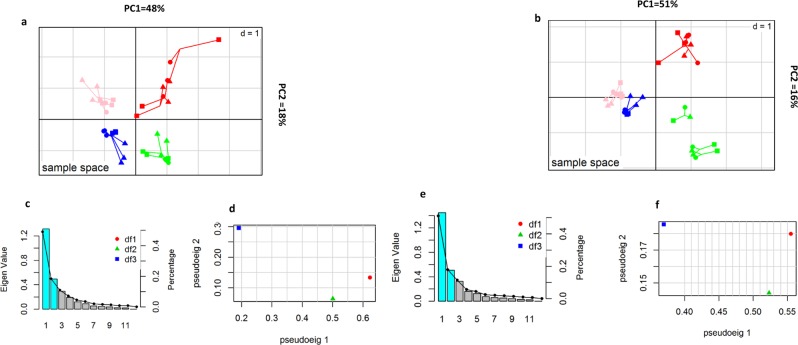
MCIA analysis of core and surface samples of cheddar cheeses. (**a,b**) The first two axes of MCIA represents metabolomics (LC/MS and GC/MS) and microbiota composition of the industrial and artisanal core (**a**) and surface (**b**) cheeses. Different shapes (df1, diamond: GC/MS data set; df2, triangle: microbiota data set; df3, square: LC/MS data set) represent the different variables connected by lines, the length of these lines is proportional to the divergence between the data. Lines for each sample are joined at a common point, at which the covariance derived from the MCIA analysis is maximal. Color shows the 4 brands of cheeses. (**c,e**) Psedo-eigenvalue space representing the percentage of variance explained by each of the MCIA component for core (**c**) and surface (**e**) datasets. (**d,f**) Pseudo-eigenvalues space of all datasets for the core (**c**) and the surface (**f**), showing overall co-structure between three datasets and shows which dataset contributes more to the total variance.

Multiple co-inertia analysis (MCIA) was used to determine whether any inter-omic relationships existed between the three datasets (16S rRNA sequences, untargeted GC-MS metabolomics and untargeted LC-MS metabolomics). Figure 3 shows the projection of industrial and artisanal cheeses onto the first two principal components (PCs) of MCIA for both core and surface data. The artisanal cheeses were separated from industrial cheeses along the horizontal axis (PC1). This clustering accounted for 48% and 51% of the variation for the core and surface cheese samples, respectively. Similarly, each brand of artisanal or industrial cheese was separated along the vertical axis (PC2) for both the core and surface cheese samples, accounting for approximately 18% and 16% of variation, respectively (Fig. 3a,b). The pair-wise *RV* (R-vector) coefficient which is multivariate generalization of the squared Pearson correlation coefficient indicated higher global similarity between GC-MS and microbiota datasets (*RV* score for core = 0.8 *RV* score for surface datasets = 0.91) when compared to the similarities between the LC-MS and microbiota datasets (*RV* score for core = 0.45, *RV* score for surface datasets = 0.77), and between the LC-MS and GC-MS datasets (*RV* score for core = 0.53, *RV* score for surface datasets = 0.81). Also, these similarities were more pronounced in the surface samples when compared to core samples.

**Figure 4:**
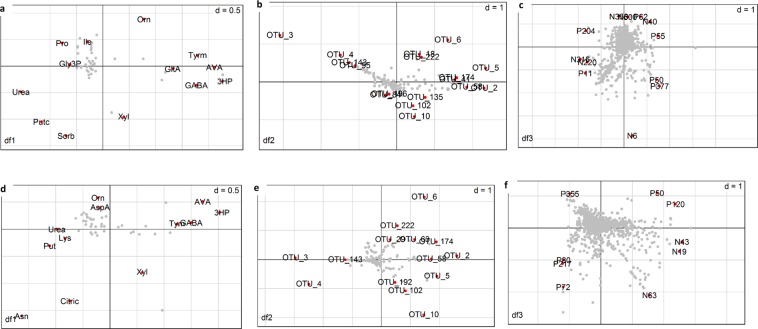
The coordination of GC/MS metabolites (**a**), OTUs (**b**), and LC/MS metabolites (**c**) for the core samples and the coordination of GC/MS metabolites (**d**), OTUs (**e**) and LC/MS metabolites (**f**) for the surface samples are shown. The OTUs and metabolites at the positive end of PC1 are associated with the artisanal cheese and at the opposite side are associated with the industrial cheeses, also these features show strongest covariance. P120: tyramine, N40: citrulline, P242: unknown peptide, P11: Proline, P155: unknown (*m/z* = 628.55, RT = 19.14), P262: Unknown (*m/z* = 631.491 = , RT = 17.80), N19: 1-formyl pentanedioic acid, P80: dipeptide (asparagine-valine), N43: unknown (*m/z* = 268.15, RT = 6.76). (See putative formula and MS2 features in Supplementary Table S3). Abbreviations for metabolites are shown in Supplementary Table S1. Identity of OTUs is shown in Supplementary Dataset S5.

The OTUs and metabolites with the strongest inter-omic covariance as predicted by MCIA are shown in Fig. 4. These OTUs and metabolites were also highly associated with the industrial (negative side of PC1) and the artisanal (positive side of PC1) cheeses. This analysis suggested OTUs assigned to *Streptococcus* and *Lactobacillus* genera and also metabolites including 3-hydroxy propanoic acid, 5-amino valeric acid, tyramine, GABA were strongly associated with artisanal cheeses for both core and surface data. Additionally, the presence of *Pediococcus* and *Kocuria* could be used to differentiate between the two artisanal cheese brands. Moreover, OTUs assigned to *Lactobacillus* and also metabolites including urea, putrescine, proline, sorbose, glycerol-3-phosphate were strongly associated with the industrial cheeses for the core samples. For the surface samples, asparagine, lysine, putrescine, urea, citrate, asparagine-valine and OTUs assigned to *Lactobacillus* and *Macrococcus* were strongly associated with the industrial cheeses.

### Correlation analysis

**Figure 5:**
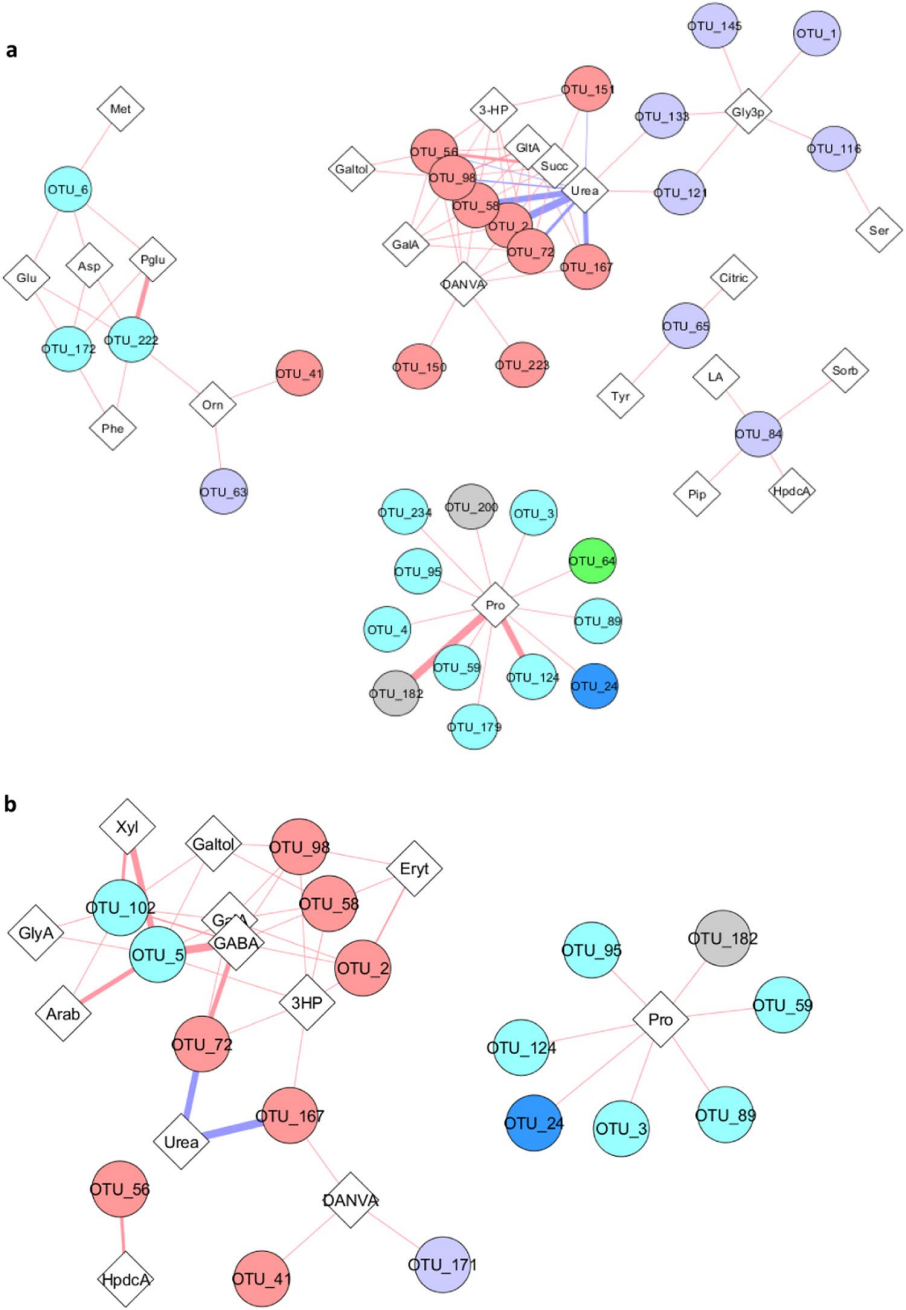
Inter-omic Spearman correlation networks. Pairwise Spearman correlation was performed for each OTU and GC/MS metabolites for the core (**a**) and the surface (**b**) samples; Any resulting correlations with q > 0.1 were removed. Red line shows positive and blue line shows negative correlation. Circles indicate taxa and diamonds indicate metabolites. Stronger correlations are shown as line thickness increases. Thicker line shows strangest correlations. Taxa keys: red nodes: *Streptococcus*, purple nodes: *Lactococcus*, cyan nodes: *Lactobacillus*, blue nodes: *Macrococcus*, green nodes: *Leuconostoc*, gray nodes*: unassigned genus*. Abbreviations for metabolites are shown in Supplementary Table S1.

Having found significant inter-omic correlations between the microbiome and metabolome of the cheeses, a Spearman analysis was used to identify the strongest contributors to the observed inter-omic correlations. An interaction network is shown in Fig. 5 to display relationships between OTUs and GC-MS metabolites. Details of significant correlations between OTUs and metabolites are shown in Supplementary Datasets S1–S4.

To identify bacterial genera with the highest number of correlations with metabolites, OTUs were binned at genus level and the number of associated (correlated) metabolites quantified. *Streptococcus*, *Lactobacillus* and *Lactococcus* displayed the highest frequencies of correlations to metabolites. *Lactococcus* showed relatively fewer correlations with metabolites when compared with the other two genera. In contrast, *Streptococcus*, *Lactobacillus*, *Macrococcus*, *Leuconostoc* and *Pediococcus* showed a greater number of correlations with metabolites in the cheese surface metabolites (LC-MS) when compared to in the cheese core. Notably, *Macrococcus* and *Leuconostoc* although both present at low relative abundances in the bacterial communities still displayed significant correlations to proline and 5 and 21 other features, respectively. Also, *Pediococcus* showed a significant correlation to a peptide, a branched chain fatty acid and 21 other features, perhaps suggesting its ability to contribute to diverse metabolites in the presence of oxygen (q < 0.1) (Supplementary Table S2).

Core cheese samples exhibited more than double the number of correlations between bacterial OTUs and amino acids than in surface samples, presumably indicating a greater level of microbial-induced peptidolysis in the cheese core (Fig. 5a; Supplementary Dataset S1). Five genera, namely, *Streptococcus*, *Lactobacillus*, *Lactococcus*, *Macrococcus* and *Leuconostoc* were significantly correlated with amines and amino acids for core samples in which *Streptococcus*, *Lactobacillus* and *Lactococcus* had excellent correlation with amino acids (*|sim-score|* > 09). However, for surface samples, only three genera *Streptococcus*, *Lactobacillus*, *Lactococcus* displayed correlations with amino acids in which *Lactobacillus* had the greatest correlations (*|sim-score|* > 09). Aromatic amino acids (phenylalanine, tyrosine), branched-chain amino acids (leucine, isoleucine and valine) and methionine are major precursors of cheese aroma compounds. Methionine was negatively correlated to *Streptococcus* (*St. salivarius*); and positively correlated to *Lactococcus* (*Lc. lactis*) on the core and the surface of cheeses, respectively. Phenylalanine showed positive correlation to *Lactobacillus* (*Lb. coryniformis*). Tyrosine, providing a bitter taste of cheeses, was positively correlated to *Lactococcus* (*Lc. lactis*). Glutamic acid, which confers umami flavour^15^ was positively correlated with *Lactobacillus (Lb. crispatus, Lb.coryniformis)*. Ornithine which is the product of arginine deiminase pathway was positively correlated to *Streptococcus* (*sim-score* > 0.9) and *Lactococcus* (*|sim-score* > 0.8). Moreover, GABA, a bioactive compound in cheese, was correlated to S*treptococcus (St. salivarius)* (*sim-score* > 0.7); and more importantly to *Lactobacillus* (*Lb. ginsenosidimutans* and *Lb.sakei*) (*sim-score* > 0.9). Putatively identified dipeptides in the cheeses were positively correlated to *Lactobacillus, Lactococcus and Macrococcus* and *Leuconostoc*; and negatively correlated with *Streptococcus*.

For hydroxylic acids and fatty acids positive correlations were found to be associated with the presence and the abundance of genera including *Lactococcus*, *Streptococcus* and *Lactobacillus*. Succinic acid providing savoury flavour in cheddar cheeses displayed positive correlations to *Streptococcus* in the core of cheeses. Furthermore, two putatively identified compounds (3-hydroxy propanoic acid and methoxy catechol) which have not been reported in cheese previously displayed significant correlations. 3-hydroxy propanoic acid was positively correlated to *Streptococcus* and *Lactobacillus* and methoxy catechol was positively correlated to *Streptococcus*.

## Discussion

By integrating three different omics datasets, this study reveals new interrelationships between the cheese-microbiota and -metabolome and identifies metabolites not previously described in cheddar cheese. This multi-omics approach coupled with integrative analysis of multiple datasets also represents a highly sensitive tool for differentiating and testing closely related foodstuffs for their provenance and authenticity. Based on the integrative analysis of three omics datasets, cheddar cheeses could be clustered according to cheese type (Fig. 3). More importantly, two highly similar industrial cheddars that were of identical age and appearance, but different brands, could also be discriminated by MCIA plots. Similarly, the two artisanal cheeses were highly distinguishable in these plots. This shows that where individual omics are unable to detect differences between cheeses, the concatenated information from multi-omics platforms can detect even fine differences. MCIA also uncovered strong relationships that existed between the cheese microbiota and metabolome, as reflected by the dataset and the correlation-based analysis of paired microbiome-metabolome datasets, and indicated specific contributors (OTUs and metabolites) to these relationships.

The composition of cheese microbiota based on sequencing of 16S rRNA-genes was broadly consistent with previously reported findings^3,16,17^. *Lactococcus*, or a combination of *Lactococcus* and *Lactobacillus*, were found to dominate the microbiota of the industrial cheddars (>90%), while *Streptococcus* (presumably *S. thermophilus*) dominated the artisanal cheeses. Although the identity of the starter cultures used and manufacturing conditions for each cheese type was not known *a priori*, it seems highly likely that these organisms would have been used as starter or adjunct cultures. However, the possibility that some of these may have come from the factory environment cannot be discounted. It is well known that flavour and other organoleptic properties of cheese are not solely determined by starter and/or adjunct cultures but also by adventitious microbiota^2^. In our study, *Pediococcus* and *Leuconostoc* (in the artisanal cheeses) and *Macrococcus* (in the industrial cheeses) were present at less than 1% but were strongly correlated to the presence and abundance of a number of cheese metabolites (Supplementary Table S2). In particular, in surface samples, *Macrococcus* was correlated to 13 metabolites including two dipeptides and an amino acid (proline), possibly due to *Macrococcus* being able to outcompete anaerobic lactic acid bacteria (LAB) in the presence of oxygen. *Pediococcus* was correlated to 21 metabolites including an unidentified peptide and a branched chain fatty acid (BCFA), 1-formyl-pentadioic acid. Organisms present in low abundance could be significant in cheese production if they have unique metabolic activities that result in the release of potent flavour compounds, and/or other compounds with implications for health. For example, *Brevibacterium* was shown to be present in low amounts in the rinds of Irish soft and hard cheeses, yet was suggested to significantly affect the flavour profiles of the cheeses^3^. Also, *Pediococcus*, normally a low-abundance component of the cheese microbiota, is known to metabolise amino acids to a variety of compounds important in quality and flavour development, including diacetyl^18^, α-aminobutyrate and alanine^19^. The association between *Pediococcus* and BCFA metabolism warrants further investigation as it could have important cheese flavour consequences. *Macrocococcus caseolyticus* is also known to contribute positively to cheese ripening, due in part to its high proteolytic activity releasing peptides (some with bioactive properties) and amino acids^20^.

Metabolic profiling using untargeted GC-MS and LC-MS revealed an extensive diversity of compounds in both industrial and artisanal cheddar cheeses. This included two compounds (3-hydroxy propanoic acid and putatively identified methylcatechol-*O-*sulfate) that, to our knowledge, have not been previously reported in any food. However, a limitation with LC-MS untargeted profiling is that the identity of many metabolites cannot be resolved due to the non-standard ionization and fragmentation that occurs during LC-MS, which makes it difficult to use or build metabolite-specific spectral databases for compound identification^21^. Therefore, in our study, relatively few compounds were able to be identified (tentatively or otherwise) using this platform. In addition, while associations between metabolites and OTUs revealed by correlation-based analyses (as used here) do not prove cause and effect, they do provide a basis for further hypothesis-based targeted studies in combination with pathway analyses.

3-hydroxy propanoic acid (3-HP) that was detected only in artisanal cheeses was positively correlated to *Lactobacillus* and *Streptococcus* (Fig. 5). This compound (3-HP) has important industrial applications, particularly as a precursor for the synthesis of a range of chemicals, and several microorganisms including *Lb. reuteri* are known to possess biochemical pathways for its production^22,23^.

A propanediol utilisation protein (PduP) has been suggested to be a key enzyme in the production of 3-HP in glycerol-fermenting microorganisms^24^. A search of the NCBI database (www.ncbi.nlm.nih.gov) showed the presence of genes putatively encoding PduP in many *Lactobacillus* species although this protein is not yet classified in the Kyoto Encyclopedia of Genes and Genomes (KEGG) database (www.genome.jp/kegg). The production of 3-HP by genetically-engineered microorganisms including *Lactobacillus*^25^ and yeast^26^, and by endophytic marine fungi^27^ has also been reported. The latter authors found that 3-HP had antimicrobial activity against *Salmonella typhi* and *Staphylococcus aureus*, two important food borne pathogens. Further studies are needed to determine the origin and significance of 3-HP in cheese (for example by nuclear magnetic resonance) where it could have a role in food protection and/or preservation.

Methoxycatechol *O*-sulphate is another compound that was detected in higher abundance in the surface of artisanal cheeses and was positively correlated with *Streptococcus* species. Genes and corresponding proteins involved in the biosynthesis of O-methoxycatechol-O-sulphate have yet to be reported. To our knowledge, this also has not been reported previously in cheese or any other food. The presence of this compound in human plasma and urine, as a result of metabolic processing of dietary phenolics, has been previously reported^28,29^, and a positive relationship between dietary polyphenols and human health is well documented. Further studies are needed to confirm the presence of this compound in cheese and its significance. The ability of some phenolic compounds originating from the catabolism of aromatic amino acids to produce off-flavours has also been observed^30^.

Other metabolites that were significantly associated with the artisanal cheeses (both in core and surface samples) were GABA, a non-protein amino acid which in cheese is associated with a sour flavour note^31^ and also has neurotransmission and antihypertensive activities^32^; tyramine, a biogenic amine frequently found in cheese as a result of tyrosine decarboxylase activity of microorganisms, and 5-amino valeric acid, a delta amino acid, rarely found in cheese and associated with negative flavour effects in hard cheese^33^. LAB, including various non-starter lactic acid bacteria (NSLAB), isolated from cheeses, have been shown to synthesize GABA from L-glutamate through glutamate decarboxylase activity^34,35^. In our study, the presence of GABA was positively correlated with both *Lactobacillus* and *Streptococcus*. Analysis of KEGG pathways for GABA formation showed multiple *Lactobacillus* and *Streptococcus* species contain genes encoding enzymes that produce GABA, including the enzyme glutamate decarboxylase [EC:4.1.1.15]. This enzyme is involved in alanine, aspartate and glutamate metabolism. Furthermore, we observed a higher relative abundance of citrulline and a lower abundance of arginine in the artisanal cheeses compared with the industrial cheeses. This was probably due to arginine deiminase pathway activity in *Streptococcus and Lactococcus*. The arginine deiminase (ADI) pathway, which is widely distributed among lactic acid bacteria, is suggested to serve as a source of energy and a mechanism for enhanced acid tolerance. However, a recent study has highlighted that ADI may not be present in *Streptococcus thermophilus*^36^.

The presence of other amino acids including lysine and proline, asparagine, arginine and various short dipeptides (asparagine-valine, threonyl-glutamate and one containing glutamate) was strongly associated with the industrial cheeses. Asparagine and lysine are biosynthetically produced following arginine and glutamate metabolism and are then catabolised to flavoursome fatty acids in cheese^37^. Higher levels of these amino acids, therefore, may contribute to the distinctive flavour profile of industrial cheeses. Free amino acids were correlated with *Lactobacillus* which is consistent with relatively high proteolytic activity of this genus compared to most other LAB. Also, reduced urea levels in the artisanal cheeses is consistent with the presence of urease-positive *Streptococcus*^38^. Accumulation of galactonic acid (an oxidised form of galactose) in artisanal cheeses is also consistent with the dominance of *S. thermophilus* since most strains of *S. thermophilus* are *gal*-negative^39^. Succinic acid, which produces umami flavour^15^, was relatively more abundant in the artisanal cheeses; it is known to be produced by citrate-fermenting strains of LAB^40^. Citrate was positively correlated with *Lactococcus*, and its relatively higher abundance in the industrial cheeses suggests that a higher proportion of the lactococci in these cheeses were non-citrate-fermenting strains. Higher relative levels of fatty acids including heptadecanoic acid and a branched chain fatty acid on the surface of artisanal cheeses could be due to lipase activity (fungal or bacterial) and/or catabolism of branched chain amino acids.

## Conclusion

This study demonstrates, for the first time, that the application of integrative analysis to multi-omics datasets could be a highly sensitive and reliable tool to distinguish between closely-related industrial and artisanal cheddar cheeses and brands thereof. Additionally, this approach helped identify new relationships between the cheese microbiota and myriads of metabolites, including those that are likely to influence product quality and sensory properties. This could also inform selection of cheese starter and/or adjunct cultures to specifically enhance cheese flavour and characteristics. Furthermore, this approach has potential application for investigating provenance, authenticity and discovering biomarkers that could be used to monitor and predict cheese quality. This provides a valuable basis for experiments for targeted studies investigating linkages between microbiota, metabolome and cheese quality, yielding key information that will be of great benefit in future cheesemaking practice. This approach could be extended further to population-level genomic (and metagenomic) studies to link metabolite production to genetic analysis of biochemical pathways within cheese microbiota.

## Materials and Methods

### Sampling

Four different types of age-matched (1 yr ± 1 month) cheddar cheeses including two industrial (large mechanised cheese manufacturer) and two artisanal brands (obtained from the super market or local producers) were analysed in this study. For each brand, triplicate individual cheeses were analysed. Cheeses were sampled aseptically both at the surface and from the inner core to allow analysis of each environment. On sampling, each cheese subsample (surface or core) was further split into two sub-samples; one of these was frozen at −80 °C for subsequent analysis of the cheese microbiota, while the second was immediately homogenized using a mortar and pestle with liquid nitrogen and then freeze-dried for subsequent metabolomics analysis.

### 16S rRNA-based cheese microbiota analysis

Total DNA was extracted from each cheese sample using a PowerSoil® DNA Isolation Kit (MO BIO Laboratories, Inc., Carlsbad, CA, USA) following the manufacturer’s instructions. The V4 region of DNA was amplified using PCR with the 16S rRNA primers 515F and 806R^41^ with cycle conditions of 95 °C for 3 min followed by 25 cycles of: 95 °C for 30 s, 55 °C for 30 s, 72 °C for 30 s and final extension at 72 °C for 5 min. Purification of the amplified 16S rRNA samples and incorporation of indices were conducted according to the 16S Metagenomic Sequencing Library Preparation instructions using Nextra^®^ XT Index Kit (Illumina, San Diego, CA, USA). A second PCR was performed to anneal indices to the 16S rRNA amplicon with the following conditions: an initial denaturation at 95 °C for 3 min, followed by 8 cycles of: 95 °C for 30 s, 55 °C for 30 s, 72 °C for 30 s and an extension at 72 °C for 5 min. A final clean-up of indexed 16S rRNA amplified samples was performed as according to the 16S Metagenomic Sequencing Library Preparation instructions (Illumina, San Diego, CA, USA). The concentrations of the purified PCR products were determined using a ND-1000 UV-Vis spectrophotometer (NanoDrop Technologies, Wilmington, DE, USA). Amplicons were pooled in equal concentrations and sequenced on an Illumina MiSeq platform (Illumina, San Diego, CA, USA).

Bacterial 16S rRNA gene sequences were analysed using a GHAP v2.1 (Greenfield Hybrid Amplicon Pipeline, developed by Paul Greenfield, 2017) which is based on USearch tools^42^. In brief, the demultiplexed reads were quality and length filtered, and then clustered at 97% similarity to generate a set of represented Operational Taxonomic Unit (OTU) sequences using USearch v8.1.1812 tools. OTU sequences were classified by using the RDP Classifier (v2.10.2)^43^ to provide a taxonomic classification for each sequence down to level of genus (where possible); and additionally, by using *Usearch-global* to find the best match with a curated set of 16S reference sequences, to provide (where possible) a species level classification. The relative abundance of each taxon in each sample was summarised using the vegan package Rv.3.4.3^44^. Analysis of similarity (ANOSIM) and Principal Component Analysis (PCA) was performed using Primer v7 (Primer-E, Plymouth, United Kingdom)^45^.

### Metabolome analysis

#### Extraction of cheddar cheese metabolites

Unless stated otherwise, all chemicals were obtained from Merck (Sydney, Australia). The protocol for metabolites extraction was carried out according to Dias *et al*.^46^ with minor modifications. Metabolites were extracted from freeze-dried cheese (60 mg) in a lysing tube containing ceramic beads (1.3 mm) and 500 μL of MeOH/H_2_O/CHCl_3_ (2.5:1:1, vol/vol/vol). Internal standards (100 μL of ^13^C_6_-sorbitol/^13^C_5_^15^N-valine in water, 0.2 mg mL^−1^) were then added to this mixture. The mixture was homogenized using a MP homogeniser (FastPrep®) (1 min, 4.5 m/s) and vortexed and incubated (37 °C for 15 min) in a thermomixer at 850 rpm. Then, the mixture was centrifuged at 13000 rpm for 15 min. The supernatant was transferred to a new Eppendorf tube, and 500 μL of MeOH/H_2_O/CHCl_3_ was added into the first lysing tube containing the previously freeze-dried sample. The samples were again vortexed and centrifuged at 13000 rpm for 15 min. The resulting supernatant was then transferred into the tube containing the original supernatant from the previous centrifugation. Pooled samples were then vortexed for 30 s and 50 μL aliquots of supernatant were transferred into separate glass inserts and dried *in vacuo* for subsequent trimethylsilyl (TMS) polar metabolite derivatisation using GC-MS and LC-MS analysis, as described below.

#### Metabolite derivatization and analysis by GC-MS

Cheese extracts were re-dissolved in 10 µL of 30 mg/mL methoxyamine hydrochloride in pyridine and derivatized at 37 °C for 120 min by mixing at 500 rpm in the presence of both 20 µL *N,O*-bis-(trimethylsilyl) trifluoroacetamide (BSTFA) and 1 µL of a retention time standard mixture [comprising 0.029% (v/v) *n*-dodecane, *n*-pentadecane, *n*-nonadecane, *n*-docosane, *n*-octacosane, *n*-dotriacontane, *n*-hexatriacontane dissolved in pyridine]. Each derivatized sample was allowed to rest for 60 min prior to injection of samples into into a GC-MS system comprised of a Gerstel PAL3 Autosampler, a 7890B Agilent gas chromatograph and a 5977B Agilent quadrupole MS (Agilent, Santa Clara, USA) using either split (1:20 split ratio) or splitless mode. The MS was adjusted according to the manufacturer’s recommendations using *tris*-(perfluorobutyl)-amine (CF43). A J&W Scientific VF-5MS column (30 m long with 10 m guard column, 0.25 mm inner diameter, 0.25 µm film thickness) was used. The injection temperature was set at 250 °C; the MS transfer line at 290 °C, the ion source adjusted to 250 °C and the quadrupole at 150 °C. Helium (UHP 5.0) was used as the carrier gas at a flow rate of 1.0 mLmin^−1^. The following temperature program was used; injection at 70 °C, hold for 1 min, followed by a 7 °C/min oven temperature, ramp to 325 °C and a final 6 min heating at 325 °C. Mass spectra were recorded at 2 scans s^−1^ with a 50–600 *m/z* scanning range.

#### Metabolite analysis by LC-MS

HPLC separation of metabolites was carried out using a Thermo Hypersil column (C18, 150 cm × 0.2 cm, 1.9 uL) on a Vanquish UHPLC system (Thermo Scientific, Waltham, MA, USA) equipped with a chilled autosampler (15 °C), heated column compartment (30 °C) and PDA (spectra collected from 190 nm to 680 nm). The mobile phase was comprised of water containing 0.1% formic acid and acetonitrile containing 0.1% formic acid. The injection volume was 3 μL. The flow rate was 0.3 mL min^−1^ with a gradient elution of 2–100% acetonitrile over 18 min with a re-equilibration time of 2 min. Analytes were detected by mass spectrometry using *Q* Exactive Plus (Thermo Scientific, Bremen, Germany), with a heated electrospray ionisation (ESI) source. The capillary temperature was 300 °C and the sheath, auxiliary and spare gases set at 28, 15 and 4 units, respectively. Source voltage was 3.6 kV for positive mode and 3.3 kV for negative mode. The mass spectrometer was operated in positive negative switching mode with full scan (90–1350 *m/z*) at 35,000 resolutions in both modes.

#### Data processing and statistical analyses of data

Chromatograms and mass spectra from GC-MS platform were processed using the Agilent MassHunter Workstation Software, Quantitative Analysis, Version B.07.01/Build 7.1.524.0. Mass spectra of eluted compounds were identified by comparison to the commercial mass spectra library NIST 08 (http://www.nist.gov), the public domain mass spectra library of Max-Planck-Institute for Plant Physiology, Golm, Germany (http://csbdb.mpimp-golm.mpg.de/csbdb/dbma/msri.html) and an *in-house* mass spectral library at RMIT University. All matching mass spectra were additionally verified by comparing their retention time to authentic standards. Resulting relative response ratios (area of analyte divided by area of the internal ^13^C_6_ –sorbitol standard and sample dry weight) for each analysed metabolite were calculated. For the metabolome datasets, zero values were replaced with half of the lowest positive value in the metabolome dataset for all analyses. Differences in the relative abundance of metabolites between the industrial and artisanal samples were validated by the Student’s *t*-test. Differences were considered significant with a *t*-test value *P* < 0.05 (*Bonferroni* corrected *P* value). If a specific metabolite had multiple trimethyl silyl derivatives the metabolite with the greater detector response and better peak shape within the dynamic range of the instrument was selected.

The MS data from the LC-MS platform were separated between positive and negative modes of ionization and converted to the mzXML format using ProteoWizard 3.0.6002 package on the MSConvert software (Proteowizard Software Foundation). Data were processed by MZmine 2.10 (MZmine 2 project) to undertake peak detection, peak filtering, chromatogram construction, chromatogram deconvolution, isotopic peak grouping, chromatogram alignment, gap filling and the search for adducts. Online databases such as the Human Metabolomics database (HMDB; http://www.hmdb.ca/) and Metabolomics Workbench (http://www.metabolomicsworkbench.org) were used to determine tentatively the identities of significant metabolites. The processed data were then subjected for further analyses in Gene Data Expressionist® (GeneData, Basel, Switzerland). PCA analysis was performed in SIMCA 15.0.1 (Umetrics AB, Umea, Sweden).

### Multiple Co-Inertia Analysis (MCIA)

A Multiple Co-Inertia Analysis (MCIA), using the language and statistical environment R together with the *omicade4* package69^14^ was performed in order to integrate multiple omics datasets where the same samples have been analysed using multiple platforms, in this case 16S rRNA gene sequence data and LC-MS metabolomic and GC-MS metabolite data. Prior to analysis, OTUs and metabolites with zero values in more than 90% of the samples were removed. MCIA is a two-step process. Firstly, a one-table ordination method (PCA) was applied to each multidimensional-omics dataset (GC/MS metabolomics, LC/MS metabolomics and 16S rRNA gene sequence dataset) to transform these into comparable lower dimensional spaces by finding axes maximising the sum of the variances of the variables. In the second step, the variance structure analyses were combined into a single analysis to find new axes on which the omics datasets could be projected by maximizing the square covariance. MCIA produces graphical outputs where different colours can be used to represent different sample datasets and different shapes can be used to represent different analysis variables (in this case bacterial OTUs and cheese metabolites). Each shape is connected by lines whose length is proportional to the divergence between the data derived from the same sample.

### Correlation analysis

All inter-omic (16S rRNA gene sequence data, and GC-MS and LC-MS based metabolomics) analysis was performed in *R*. Microbiota and metabolomics data were thresholded such that OTUs and metabolites, which are present in less than three samples, were removed. The reasoning for this cut-off, which was applied separately to core and surface samples, was to avoid the effects of significant inter-omic correlations being enriched due to rare taxa and metabolites. Correlations were computed using the *CCREPE* (Compositionality corrected by renormalization and permutation package) *R* package (v.22.1.0) (http://huttenhower.sph.harvard.edu/ccrepe)^47^. *CCREPE* provides compositionality-corrected *p*-values, *q*-values and the N-dimensional checkboard score (NC-Score), which is a novel similarity measure, for all correlations between two datasets. The results were filtered to remove non-statistically significant relationships (q-value < 0.1). Cytoscape 3.6.0 was used to generate correlation networks^48^.

## Supplementary information

Supplementary information.

Dataset 1.

Dataset 2.

Dataset 3.

Dataset 4.

Dataset 5.

## Data Availability

The datasets generated during and/or analysed during the current study are available from the corresponding author upon reasonable request.
