# Author Correction: New insights into cheddar cheese microbiota-metabolome relationships revealed by integrative analysis of multi-omics data

**DOI:** 10.1038/s41598-021-82097-4

**Published:** 2021-01-25

**Authors:** Roya Afshari, Christopher J. Pillidge, Elizabeth Read, Simone Rochfort, Daniel A. Dias, A. Mark Osborn, Harsharn Gill

**Affiliations:** 1 School of Science, RMIT University, Bundoora, PO Box 71, Bundoora, VIC 3083 Australia; 2 School of Health and Biomedical Sciences, RMIT University, Bundoora, PO Box 71, Bundoora, VIC 3083 Australia; 3 Biosciences Research Division, Department of Environment and Primary Industries, AgriBiosciences, 5 Ring Road, Bundoora, VIC 3083 Australia

Correction to: *Scientific Reports* https://doi.org/10.1038/s41598-020-59617-9, published online 21 February 2020

This Article contains an error in Figure 1, where the image used in Figure 1c is a duplication of Figure 1b. The correct Figure 1 appears below:

**Figure 1:**
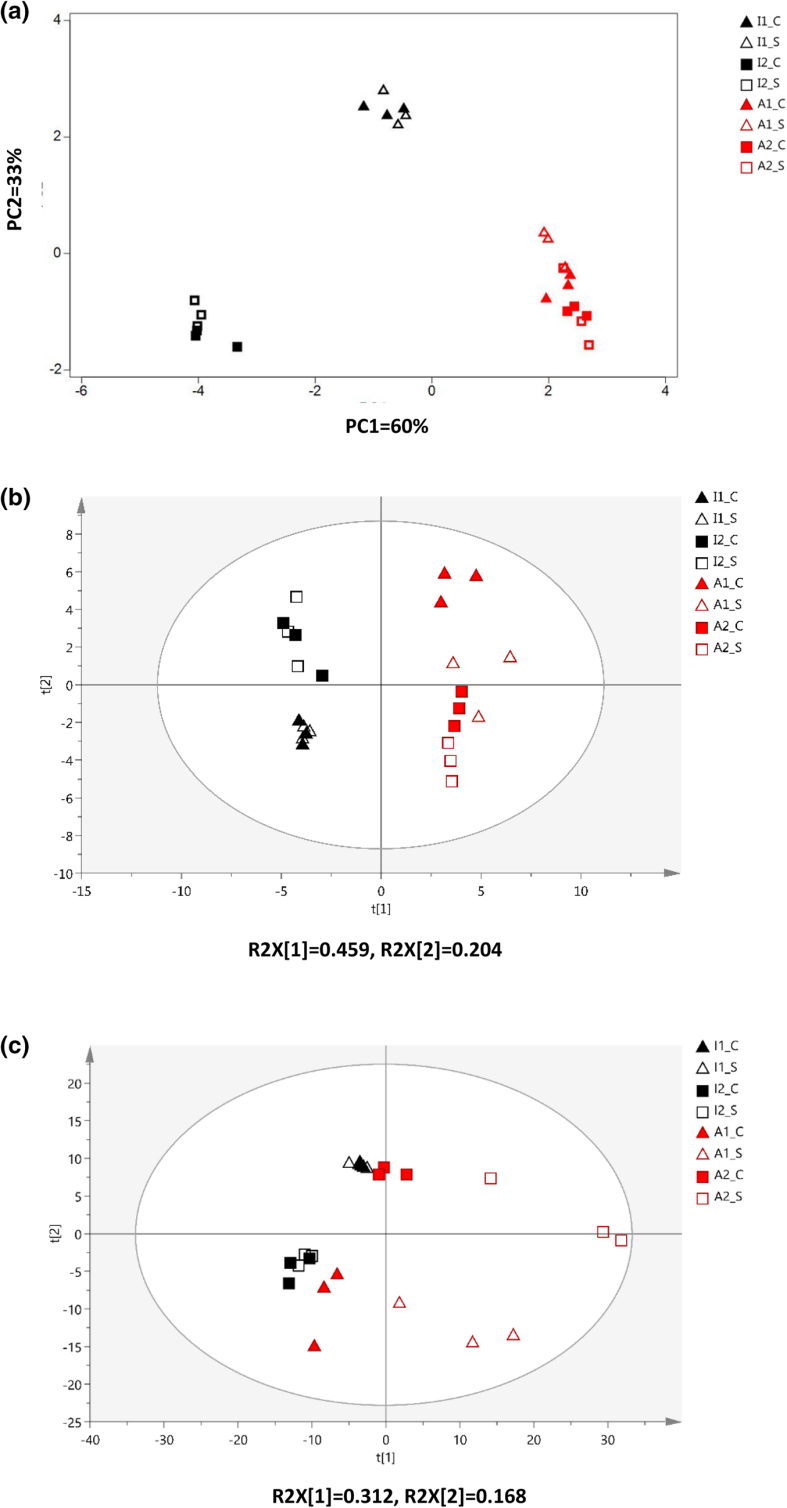
Principal component analysis (PCA) of industrial and artisanal cheeses for (**a**) microbiota, (**b**) GC-MS metabolites and (**c**) LC-MS metabolites. I: industrial, A: artisanal; C: core, S: Surface; number indicates different brand for each artisanal and industrial cheese.

