# Supplementary material for: New insights into cheddar cheese microbiota-metabolome relationships revealed by integrative analysis of multi-omics data
Source: Sci Rep. 2020 Feb 21;10:3164. doi: 10.1038/s41598-020-59617-9 (PMC7035325; doi:10.1038/s41598-020-59617-9)
Supplement: Supplementary file 1 — Supplementary information. [file 41598_2020_59617_MOESM1_ESM.pdf]

New insights into cheddar cheese microbiota-metabolome relationships revealed by integrative analysis of multi-omics data

Roya Afshari[a], Christopher J. Pillidge[a], Elizabeth Read[c], Simone Rochfort[c], Daniel A. Dias[b], Andrew Mark Osborn[a], Harsharn Gill[a] *

[a] School of Science, RMIT University, Bundoora, PO Box 71, Bundoora VIC 3083, Australia

[b] School of Health and Biomedical Sciences, RMIT University, Bundoora, PO Box 71, Bundoora VIC 3083, Australia

[c] Biosciences Research Division, Department of Environment and Primary Industries, AgriBiosciences, 5 Ring Road, Bundoora, Victoria VIC 3083, Australia

*Corresponding author. Email: harsharn.gill@rmit.edu.au Tel: +61(3)9925 2600

This file includes:

Supplementary tables (Table S1-Table S3)

Other supplementary information for this manuscript includes the following:

Supplementary dataset 1 as an Excel file: Supplementary_Dataset_S1.xls

Supplementary dataset 2 as an Excel file: Supplementary_Dataset_S2.xls

Supplementary dataset 3 as an Excel file: Supplementary_Dataset_S3.xls

Supplementary dataset 4 as an Excel file: Supplementary_Dataset_S4.xls

Supplementary dataset 5 as an Excel file: Supplementary_Dataset_S5.xls

Supplementary table legends

Table S1. Response ratios of metabolites in artisanal cheese compared to industrial cheese. For industrial cheese, response ratios are set at unity with associated standard error between replicates showing the overall variance. For artisanal cheese, response ratios are presented relative to those of industrial cheeses (i.e. as fold differences). Data were log transformed prior to statistical analysis by t-test.

Table S2. Genera with significant correlations (q<0.1) to metabolites (GC-MS and LC-MS) in core and surface samples

Table S3. Mass to charge ratio, retention time and MS2 features of putatively identified metabolites

Table S1. Response ratios of metabolites in artisanal cheese compared to industrial cheese. For industrial cheese, response ratios are set at unity with associated standard error between replicates showing the overall variance. For artisanal cheese, response ratios are presented relative to those of industrial cheeses (i.e. as fold differences). Data were log transformed prior to statistical analysis by t-test.

Metabolites	Abbreviations	Core		Surface	
**Amino acids and amines**		**IND**	**ART**	**IND**	**ART**
5-Aminovaleric	DANVA	ND	-	ND	-
Tyramine	Tym	ND	-	ND	-
GABA	GABA	ND	-	ND	-
Putrescine	Put	1.00 ± 0.13	0.69 ± 0.46	1.00 ± 0.15	0.31 ± 0.61
Tyrosine	Tyr	1.00 ± 0.25	1.029 ± 0.39	1.00 ± 0.25	0.62 ± 0.33
Lysine	Lys	1.00 ± 0.22	0.47 ± 0.24	1.00 ± 0.16	0.13 ± 0.49**
Glycine	Gly	1.00 ± 0.35	1.17 ± 0.61	1.00 ± 0.39	0.55 ± 0.36
Valine	Val	1.00 ± 0.25	1.25 ± 0.21	1.00 ± 0.28	0.72 ± 0.31
Serine	Ser	1.00 ± 0.12	0.86 ± 0.52	1.00 ± 0.14	1.73 ± 0.64
Leucine	Leu	1.00 ± 0.20	1.3 ± 0.13	1.00 ± 0.32	0.87 ± 0.29
Isoleucine	Ile	1.00 ± 0.35	1.61 ± 0.34	1.00 ± 0.38	0.80 ± 0.43
Threonine	Thr	1.00 ± 0.35	2.75 ± 0.34	1.00 ± 0.19	4.88 ± 0.77
Proline	Pro	1.00 ± 0.40	0.19 ± 0.53	1.00 ± 0.44	0.38 ± 0.55
Phenylalanine	Phe	1.00 ± 0.26	2.03 ± 0.34	1.00 ± 0.30	1.22 ± 0.52
Alanine	Ala	1.00 ± 0.23	2.33 ± 0.46	1.00 ± 0.26	0.73 ± 0.36
Piperidine	Pip	1.00 ± 0.13	0.73 ± 0.07	1.00 ± 0.13	0.37 ± 0.29*
Asparagine	Asn	1.00 ± 0.20	0.34 ± 0.30	1.00 ± 0.10	0.06 ± 0.73*
Urea	Urea	1.00 ± 0.09	0.03 ± 0.61*	1.00 ± 0.09	0.06 ± 0.17**
Uracil	Uracil	1.00 ± 0.18	1.78 ± 0.12*	1.00 ± 0.20	1.28 ± 0.25
Aspartate	AspA	1.00 ± 0.38	4.11 ± 0.42	1.00 ± 0.38	2.79 ± 0.44
Glutamate	Glu	1.00 ± 0.29	2.25 ± 0.37	1.00 ± 0.32	1.09 ± 0.54
Methionine	Met	1.00 ± 0.27	3.12 ± 0.42	1.00 ± 0.23	4.40 ± 0.63
**Fatty acids**					
Pentadecanoic acid	PDA	1.00 ± 0.19	1 ± 0.05	1.00 ± 0.15	19.65 ± 0.43*
Heptadecanoic acid	HepA	1.00 ± 0.23	0.97 ± 0.05	1.00 ± 0.19	33.28 ± 0.42**
Decanoic acid	DecA	1.00 ± 0.05	1.5 ± 0.17	1.00 ± 0.12	2.66 ± 0.39*
Palmitic acid	PA	1.00 ± 0.10	1.69 ± 0.21	1.00 ± 0.13	11.81 ± 0.42*
Stearic acid	STA	1.00 ± 0.08	1.7 ± 0.32	1.00 ± 0.12	7.71 ± 0.36*

Cholesterol	Cholesterol	1.00	±	0.07	1.4	±	0.08*	1.00	±	0.07	1.18	±	0.15
**Organic acids**													
3-hydroxypropanoic acid	3HP		ND			-			ND			-	
Glutaric acid	Glt		ND			-			ND			-	
Lactic acid	Lac	1.00	±	0.12	1.53	±	0.18	1.00	±	0.15	0.88	±	0.2*
Oxalic acid	Oxal	1.00	±	0.11	1.1	±	0.05	1.00	±	0.05	0.80	±	0.30*
Succinic acid	Succ	1.00	±	0.27	8.87	±	0.10	1.00	±	0.28	3.35	±	0.30*
Glyceric acid	GlyA	1.00	±	0.17	1.98	±	0.10*	1.00	±	0.2	2.04	±	0.15
Glutamic acid	Glu	1.00	±	0.26	643	±	0.22	1.00	±	0.32	1.09	±	0.54
Malonic acid	MalA	1.00	±	0.30	1.40	±	0.38	1.00	±	0.31	0.81	±	0.32
Hydroxyglutaric acid	Hglt	1.00	±	0.30	1.46	±	0.18	1.00	±	0.34	0.76	±	0.21
Citric acid	Citric	1.00	±	0.17	0.95	±	0.15	1.00	±	0.08	1.43	±	0.74
Galactonic acid	GalA		ND			-			ND			-	
Pyroglutamic acid	Pglu	1.00	±	0.26	2.22	±	0.37	1.00	±	0.29	0.90	±	0.36
**Sugars and sugars phosphates**													
Erythritol	Eryt	1.00	±	0.40	31.4	±	0.40*	1.00	±	0.45	15.57	±	0.26*
Arabitol	Arab	1.00	±	0.98	3.94	±	0.64	1.00	±	0.98	21.77	±	0.43**
**Xylitol**	Xyl	1.00	±	0.06	227.16	±	0.63	1.00	±	0.04	174.21	±	0.65*
Glycerol-3-phosphate	Gly3P	1.00	±	0.07	0.31	±	0.15**	1.00	±	0.03	0.73	±	0.07*
Galactitol	Galtol	1.00	±	0.66	24.81	±	0.21*	1.00	±	0.71	6.05	±	0.23*
Inositol myo	MI	1.00	±	0.04	2.11	±	0.080**	1.00	±	0.03	1.10	±	0.14

Data represents mean of 3 representatives samples +/- standard error. ** shows a *t*-test value $P < 0.05$ / (number of metabolites) while * shows a *t*-test value between this *P* value and 0.05 (i.e. below 0.05, but not below the Bonferroni corrected. ND: not detected in industrial cheeses and was significantly higher in artisanal cheeses *P value* <0.0001.

Table S2. Genera with significant correlations (q<0.1) to metabolites (GC-MS and LC-MS) in core and surface samples.

	**Relative abundance (%)**		**LC/MS**		**GC/MS**	
Genus	Core	Rind	Core interactions, number	Rind interactions, number	Core interactions, number	Rind interactions, number
Streptococcus	42.28	37	18	92	8	8
Lactobbacillus	22.20	13.1	24	135	7	8
Lactococcus	33.90	49.1	16	23	10	1
Macrococcus	0.02	0.02	4	13	1	0
Leuconostoc	0.008	0.002	0	5	1	0
Pediococcus	0.1	0.35	0	21	0	0

Table S3. Mass to charge ratio, retention time and MS2 features of putatively identified metabolites.

M/Z	RT	Charge	Formula (neutral)	putative identity	MS2 features
232.1293	1.28	$[M+H]^+$	$C_9H_{17}N_3O_4$	ASN-VAl	$C_9H_{18}O_4N_2$, $C_9H_{15}O_4N_2$, $C_8H_{13}O_2N_2$, $C_4H_9O_3N_2$, $C_6H_{13}NO_2$ $C_3H_7ON_2$, $C_4H_{10}N$
146.1177	1.27	$[M+H]^+$	$C_7H_{15}O_2N$	gamma-Butyrobetaine	$C_7H_{16}NO_2$, $C_6H_{12}NO_2$, $C_4H_{10}NO_2$, $C_5H_{10}NO$, $C_5H_{10}N$, $C_3H_{10}N$
146.117	1.27	$[M+H]^+$	C_7 H_{15} O_2 N	gamma-Butyrobetaine	$C_7H_{16}NO_2$, C_6 $H_{12}NO_2$, $C_4H_{10}NO_2$, $C_5H_{10}NO$, $C_5H_{10}N$, $C_3H_{10}N$
174.087	1.19	$[M+H]^+$	$C_6H_{13}O_3N_3$	Citrulline	$C_6H_{12}O_3N_3$, $C_6H_9O_3N_2$ $C_5H_{11}O$ N_2, $C_5H_8O_2N$
173.1	1.15	$[M-H]^-$	$C_6H_{14}O_2N_4$	*Arginine	$C_6H_{15}N_4O_2$, $C_6H_{12}N_3O_2$, $C_5H_{12}ON_3$
199.060	3.12	$[M-H]^-$	$C_9H_{12}O_5$	1-Formyl-1-propenyl) pentanedioic acid	C9H11O5, C9H9O5, C9H9O4
231.134	2.014	$[M-H]^-$	$C_8H_{18}O_3N_5$	unknown	
258.145	1.97	$[M-H]^-$	C_{11} $H_{21}O_4N_3$	Di peptide containing glutamate	$C_{11}H_{20}$ O_4N_3, $C_{11}H_{18}$ O_3N_3, $C_{11}H_{16}O_2N_3$, $C_8H_{13}O_2N_2$
247.093	1.27	$[M-H]^-$	$C_9H_{16}O_6N_2$	Threoninyl-Glutamate	$C_9H_{15}N_2O_6$, $C_9H_{13}N_2O_5$, $C_7H_9N_2O_4$, $C_6H_{11}N_2O_3$, $C_6H_9N_2O_2$, $C_5H_6NO_3$
242.012	3.51	$[M-H]^-$	-	Unknown	-
203.001	5.28	$[M-H]^-$	$C_7H_8O_5S$	O-methoxycatechol-O-sulphate	$C_7H_7O_5S$, $C_7H_7O_2$
103.038	2.65	$[M-H]^-$	$C_2H_6O_2N_3$	Unknown	$C_2H_5O_2N_3$, $C_2H_3O_2N_3$, C_2H_3N3O, CH_3N_3
187.108	1.99	$[M-H]^-$	-	-	ND

M/Z	RT	Charge	Formula (neutral)	putative identity	MS2 features
119.025	15.53	$[M+H]^+$	-	-	ND
214.05	1.18	$[M-H]^-$	-	-	ND
279.16	10.92	$[M-H]^-$	-	-	ND
361.20	11.8	$[M-H]^-$	-	-	ND
